# Digital maternity care in Germany: a cross-sectional web-based survey on midwives’ perceptions

**DOI:** 10.1007/s00404-025-08239-5

**Published:** 2026-01-20

**Authors:** Sebastian Griewing, Pia Teske, Johannes Wichmann, Zoe Oftring, Johannes Knitza, Corinna Keil, Nikolas Tauber, Jule Däumichen, Marc Potthast, Stephanie Wallwiener, Uwe Wagner, Markus Wallwiener, Niklas Gremke, Michael Leyer, Hanna Gehling, Sebastian Kuhn

**Affiliations:** 1 Institute for Digital Medicine, Department of Medicine, Philipps-University Marburg, Marburg, Germany; 2 Midwifery Science, Department of Health Sciences, Fulda University of Applied Sciences, Fulda, Germany; 3 Clinic of Obstetrics and Gynecology, Department of Medicine, Philipps-University Marburg, University Hospital Giessen and Marburg, Campus Marburg, Marburg, Germany; 4 Research Group Digitalization and Process Management, Department of Economics, Philipps-University Marburg, Marburg, Germany; 5 Clinic of Pediatrics, Department of Medicine, Philipps-University Marburg, University Hospital Giessen and Marburg, Campus Marburg, Marburg, Germany; 6 Clinic of Obstetrics and Gynecology, Department of Medicine, University Hospital Schleswig-Holstein, Campus Luebeck, Luebeck, Germany; 7 Clinic of Obstetrics and Gynecology, Department of Medicine, Martin-Luther-University Halle-Wittenberg, Halle, Germany; 8 Commission Digital Medicine, German Society of Gynecology and Obstetrics, Berlin, Germany

**Keywords:** Digital health, Telemedicine, Artificial intelligence, Midwifery, Obstetrics

## Abstract

**Purpose:**

Maternity care is a central component of any healthcare system and is largely provided by midwives. Considering increasing cost pressures and growing demand for efficiency within the German healthcare system, the development of efficient, digitally supported care models are both encouraged and actively promoted, especially in pregnancy. However, to date, no such model has been sustainably established in the field of maternity care. In particular, the perspectives of midwives have largely been neglected.

**Methods:**

As part of an initial needs assessment for the Participatory Design of a digitally supported maternity care model, this study uses a cross-sectional web-based questionnaire to explore midwives’ perceptions of their current work situation, use of digital tools and digital pregnancy care.

**Results:**

92.2% of participants (n = 129) perceive increasing strain on maternity care in Germany (5-point Likert; M = 4.49, SD = ± 0.69). 87.6% use a variety of digital tools in their professional environment, yet unvalidated and unauthorized solutions. Self-perceived digital competence is high (10-point NRS; 7.09 ± 1.48). The intention to use the technology decreases in parallel with the level of awareness, being highest for the electronic patient record (5-point Likert; 72.1%; 3.84 ± 0.97) and lowest for artificial intelligence (38.8%; 3.17 ± 1.05).

**Conclusion:**

The study highlights midwives’ openness to digital solutions, their active, though informal, use of such tools, and emphasizes the need to integrate their perspectives into the development of certified, sustainable digital care models in maternity care within an increasingly strained healthcare system.

**Supplementary Information:**

The online version contains supplementary material available at 10.1007/s00404-025-08239-5.

## Introduction

In the past 10 years, around 750,000 children have been born in Germany every year [1]. The German Social Code guarantees pregnant individuals the right to be cared for by a midwife during pregnancy, birth, postpartum, and breastfeeding (§24d Sozialgesetzbuch SGB V). This midwife support is provided by around 27,000 midwives [2]. In recent times, care shortages have repeatedly led to a debate on how midwifery care can be sustainably strengthened. In 2020, the new Midwifery Act (Hebammengesetz, HebG) and its connected Midwifery Study and Examination Decree (Hebammenstudien- und Prüfungsverordnung, HbStPrV) have announced midwifery competencies in science-based planning, organization, implementation, management, and evaluation of even highly complex care processes. Here, it is claimed that midwives should use digital skills, research-based problem-solving, and new technologies to shape a cost-effective, efficient, and high-quality midwifery practice (HebStPrV, Appendix 1, Competence area II).

International evidence across a range of healthcare contexts, including low- to high-resource settings, and both rural and urban regions, suggests that digital care services can effectively complement traditional maternity care [3–5]. In Germany, however, progress in the digital transformation of maternal health services has been comparatively low [6]. In accordance with the German maternity guidelines (Mutterschafts-Richtlinie, Mu-RL), antenatal care relies on sequential in-patient visits with the caring gynecologist and written documentation in the so-called mother pass (Mutterpass), a physical booklet introduced to German pregnancy care in 1963 [7].

Despite the increasing availability of commercially developed digital health applications, which often lack clinical validation, no certified digital maternity care programs have yet been integrated into the statutory health insurance system [8]. This situation persists even though the passage of the Digital Care Act (Digitale-Versorgungs-Gesetz, DVG) in 2019 established a legal framework for the reimbursement of prescribable digital health applications (DiGA) [9]. As a result, three applications are currently approved in the field of women’s health in the areas of breast cancer therapy and endometriosis, but none in maternity care [8].

Furthermore, the German Federal Ministry of Health (Bundesministerium für Gesundheit, BMG) has articulated its commitment to digitally supported care models within its national Digitalization Strategy in 2023 [10]. A common example of the structured process for researching, developing and implementing digitally supported care programs in Germany can be found for heart failure patients in the specialty of cardiology [11]. This has enabled the transition from sequential in-person care to digitally supported continuous prophylaxis and risk monitoring for heart failure patients, which has been shown to significantly reduce disease-associated morbidity and mortality [12]. Building upon this, the German Federal Ministry of Health sets the establishment of person-centered and digitally supported care processes as a strategic field of action for the ongoing decade [10]. Beyond that, the BMG formulates particular potential for digital care concepts in relation to pregnancy [7].

This study is part of a broader scientific project aimed at the development, evaluation, and validation of a digitally supported maternity care model. Specifically, to inform the participatory design of a certified digital maternity care model, we first sought to characterize midwives’ workload, current use of digital tools and readiness for future technologies in a cross-sectional, non-probabilistic convenience sample.

## Methods

### Study design

This study constitutes the initial step within the needs assessment phase of a structured participatory design (PD) process, following the framework outlined by Clemensen et al. [13]. The overarching objective of this PD process is to inform the development of a digitally supported maternity care model within the German healthcare system.

Participatory design is an established method that promotes the participation of potential users in the development and implementation process of health technologies. PD is an inherently iterative process in which each phase is planned based on the results of the previous phase, taking into account the input of key stakeholder groups. Although alternative approaches, such as co-design, the development life cycle, and user-centered design, are also employed in health technology assessment, PD has demonstrated particular utility in European healthcare contexts. Notably, in Denmark, PD has been instrumental in the successful deployment of digital health interventions across a range of clinical domains, including the remote monitoring of preterm infants [14–16].

The PD methodology typically unfolds in four sequential phases: (1) a needs assessment, (2) design and development, (3) testing and retesting, and (4) the comprehensive clinical evaluation of the final system. To accommodate the diverse information requirements across these phases, PD employs a mixed-methods research approach.

**Fig. 1:**
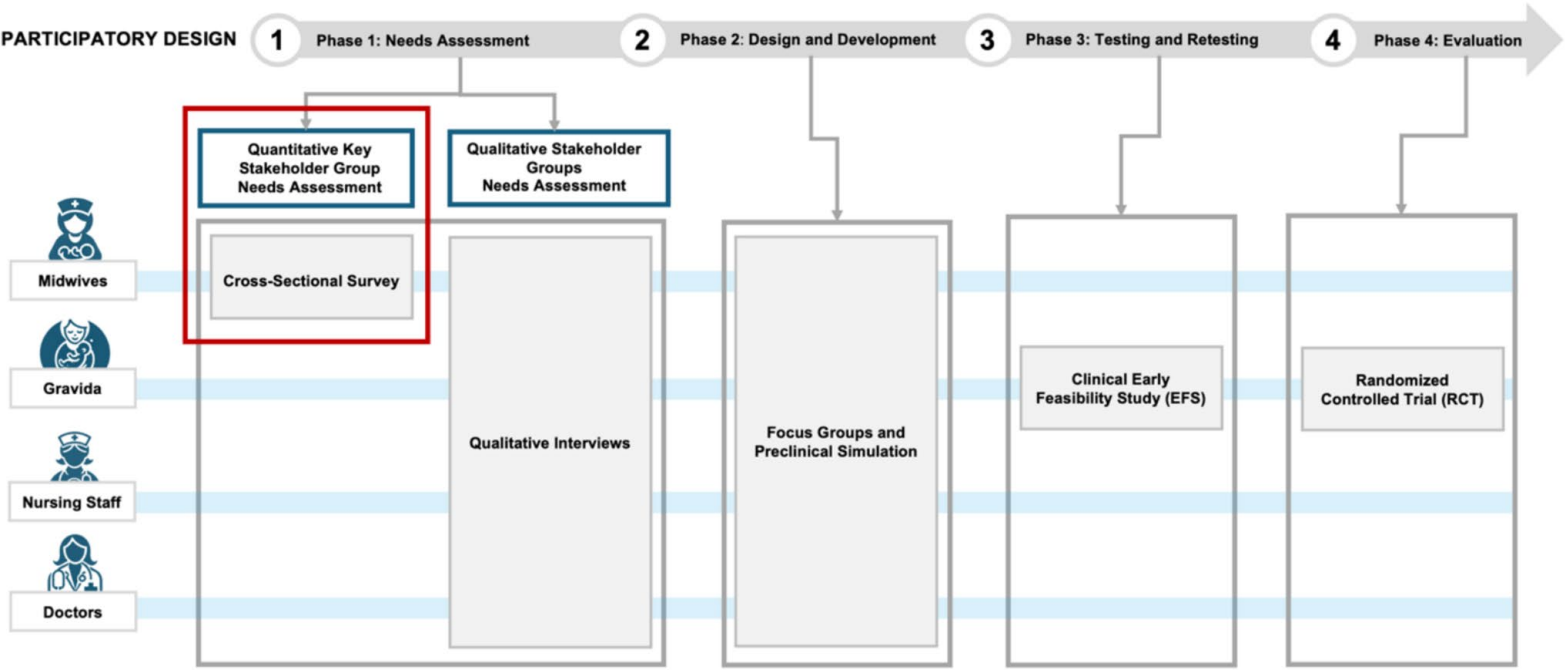
Study design (marked in red) and embedding in the participatory design process

This study represents the first component of the initial needs assessment phase, see Fig. 1. Specifically, it involves a cross-sectional survey designed to gather empirical data from a non-probabilistic convenience sample on midwives’ and midwifery students’ experiences, needs and attitudes regarding their current work environment and the integration of digital technologies into maternity care. For this reason, the present research was conducted as a cross-sectional web-based survey.

### Questionnaire, data collection and data analysis

The survey was designed, conducted, and reported in accordance with the CHERRIES (Checklist for Reporting Results of Internet E-Surveys) checklist [17]. The questionnaire covered four thematic sub-sections: (1) perception of the current work situation, (2) use of digital tools in the professional environment, (3) perception of digital maternity care and self-rated digital competence, and (4) basic demographic data. Items were generated based on a review of the literature on digital health [18–21], technology acceptance research [22–29], as well as conceptual considerations derived from the PD framework described by Clemensen et al. [13]. We considered existing validated instruments for digital literacy and technology acceptance; however, no single tool was identified that captured the range of technologies of interest (electronic patient record, electronic maternity record, telemonitoring, artificial intelligence) in the specific context of German maternity care while remaining feasible for use in a short web-based survey. For this reason, we adapted a concise context-specific instrument.

The draft questionnaire underwent internal review for clarity, relevance and content validity by two digital health researchers and one practicing midwife. Subsequently, it was piloted with five midwives and midwifery students to assess usability, language, and technical functionality. Minor adjustments were made to wording and response options based on this feedback. The final questionnaire included a total of 30 items. Measurement was facilitated by either 5-point Likert scale (strongly disagree–strongly agree), multiple choice, 10-point numeric rating scale (NRS 1–10) or in binary choice (yes/no) format. The original German version of the survey can be found in the supplementary material; see Supplementary 1.

The survey was viewed 444 times, started by 261 individuals, and completed in full by 129 respondents, corresponding to a participation rate of 58.8% and a completion rate of 29.1% when calculated according to CHERRIES recommendations. Only fully completed questionnaires were included in the analysis. As all items were mandatory once the questionnaire was started, there were no item-level missing data among included respondents. We acknowledge that the exclusion of responses may introduce attrition bias, as individuals who discontinued the survey may differ systematically from completers.

Data collection was conducted using an open, primarily anonymized, web-based questionnaire hosted on the Unipark survey platform (Tivian XI GmbH, Cologne, Germany). Technical measures to prevent multiple submissions included the use of browser cookies and time-stamped response logs. IP addresses and other metadata were not stored. The dataset was screened for implausible duplicates, e.g., identical time stamps or extremely short completion times, but no suspected duplicate entries were identified. Data were stored on servers compliant with General Data Protection Regulation (GDPR) and German Datenschutz-Grundverordnung (DSGVO).

Recruitment followed a non-probabilistic convenience-sampling approach. The questionnaire was distributed to midwives and midwifery students across Germany via QR codes and a URL link through direct outreach at the German Midwifery Congress (May 5–7, 2025, in Münster, Germany), as well as through national and regional professional networks, including the Commission Digital Medicine of the German Society of Gynecology and Obstetrics, and midwifery networks (email lists and local distribution by members of the respective professional organizations). This recruitment strategy likely favored midwives and midwifery students who are more professionally networked and more digitally engaged than the broader midwifery workforce.

Data analysis was conducted in IBM SPSS Statistics (Version 29.0.2.0 (20); IBM Corporation, Armonk, USA). In line with the exploratory aims of this initial needs assessment as part of a broader PD process, analyses were purely descriptive. We calculated absolute frequencies, arithmetic means (M), standard deviations (SD), and 95% confidence intervals (CI) for the survey items. No inferential hypothesis testing was performed (Table 1).

**Table 1:** Overview of the questionnaire parts, items and measurement methods

Thematic sub-sections	Questionnaire Part	Items	Measurement
1	Work situation	1.1 I have enough time to look after the pregnant women/women who have recently given birth 1.2 I am satisfied with my current work situation in midwifery/obstetrics 1.3 I have the feeling that the provision of midwifery/obstetrics in Germany is under increasing pressure 1.4 I would like to reduce my average working hours	5-point Likert 5-point Likert 5-point Likert 5-point Likert
2	Use of digital tools	2.1 Do you use the following electronic devices in your work environment? 2.2 Do you use the following social networks in your work environment? 2.3 Do you use digital messengers in your work environment? 2.4 What do you use digital messengers for in your work environment? 2.5 Please rate the following statement: I have concerns about using digital messengers in my work environment 2.6 I use digital messengers in my work environment due to a lack of alternatives	Multiple choice Multiple choice Multiple choice Multiple choice 5-point Likert Binary choice
3	Perception of digital pregnancy care	**Electronic patient record (ePA)** 3.1 I am aware of the electronic patient record (ePA) 3.2 I have already used the electronic patient record (ePA) **Electronic maternity record (eMutterpass)** 3.3 I am aware of the electronic maternity record (eMutterpass) 3.4 I have already used the electronic maternity record (eMutterpass) **Telemonitoring (TM)** 3.5 I am aware of the concept of telemonitoring from other areas of healthcare (for example, heart failure monitoring in cardiology) 3.6 I am aware of the concept of telemonitoring from midwifery/obstetrics 3.7 I have already used telemonitoring in midwifery/obstetrics **Artificial intelligence (AI)** 3.8 I am aware of the use of artificial intelligence from other areas of healthcare (for example, skin cancer screening in dermatology) 3.9 I am aware of the use of artificial intelligence in midwifery/obstetrics 3.10 I have already used artificial intelligence in midwifery/obstetrics **Intention-to-use in midwifery/obstetrics** 3.11 I can imagine using the electronic patient record (ePA) in the field of midwifery/obstetrics in the future 3.12 I can imagine using the electronic maternity record (eMutterpass) in the field of midwifery/obstetrics in the future 3.13 I can imagine using telemonitoring in the field of midwifery/obstetrics in the future 3.14 I can imagine using artificial intelligence in the field of midwifery/obstetrics in the future **Digital competence** 3.15 How would you rate your own digital competence on a scale from 1 to 10? (1 = very low, 10 = very high) 3.16 How would you rate the need for training to improve your digital competence on a scale from 1 to 10? (1 = very low, 10 = very high)	Binary choice Binary choice Binary choice Binary choice Binary choice Binary choice Binary choice Binary choice Binary choice Binary choice 5-point Likert 5-point Likert 5-point Likert 5-point Likert NRS 1–10 NRS 1–10
4	Basic demographic data	4.1 Age (in years) 4.2 Gender (male, female or diverse) 4.3 Professional title (head midwife, midwife, midwifery student, teaching midwife, advanced practice midwife) 4.4 Years of experience (in years (y): < 2 y, 2–5 y, 5–10 y, 10–20 y, 20–30 y, > 30 y, currently still studying)	

## Results

### Study population

Mean age was 30.7 years with a minimum of 19 and a maximum of 64 years. The study population splits into 48.1% midwifery students and 51.9% fully trained midwives; their work experience ranges from less than 2 years (20.9%) up to more than 30 years (17.9%) (Table 2).

**Table 2:** Characteristics of study population (n = 129)

Age in years (Item 4.1)	
M (± SD)	30.7 (± 12.1)
Min	19
Max	64
Gender (Item 4.2)	
Female, n (%)	126 (97.7%)
Male, n (%)	0 (0%)
Non-binary, n (%)	3 (2.3%)
Job title (Item 4.3)	
Midwife^a^, n (%)	67 (51.9%)
Midwifery student, n (%)	62 (48.1%)
Years of experience (midwives only) (Item 4.4)	
< 2 y	20.9% (14)
2–5 y	25.4% (17)
5–10 y	19.4% (13)
10–20 y	6.0% (4)
20–30 y	10.4% (7)
> 30 y	17.9% (12)

n, 129; M, arithmetic mean; SD, standard deviation; Min, minimum; Max, maximum, y, years

^a^thereof: 9.0% head midwife, 67.2% midwife, 19.4% teaching midwife, 4.5% advanced practice midwife

### Work situation

**Fig. 2:**
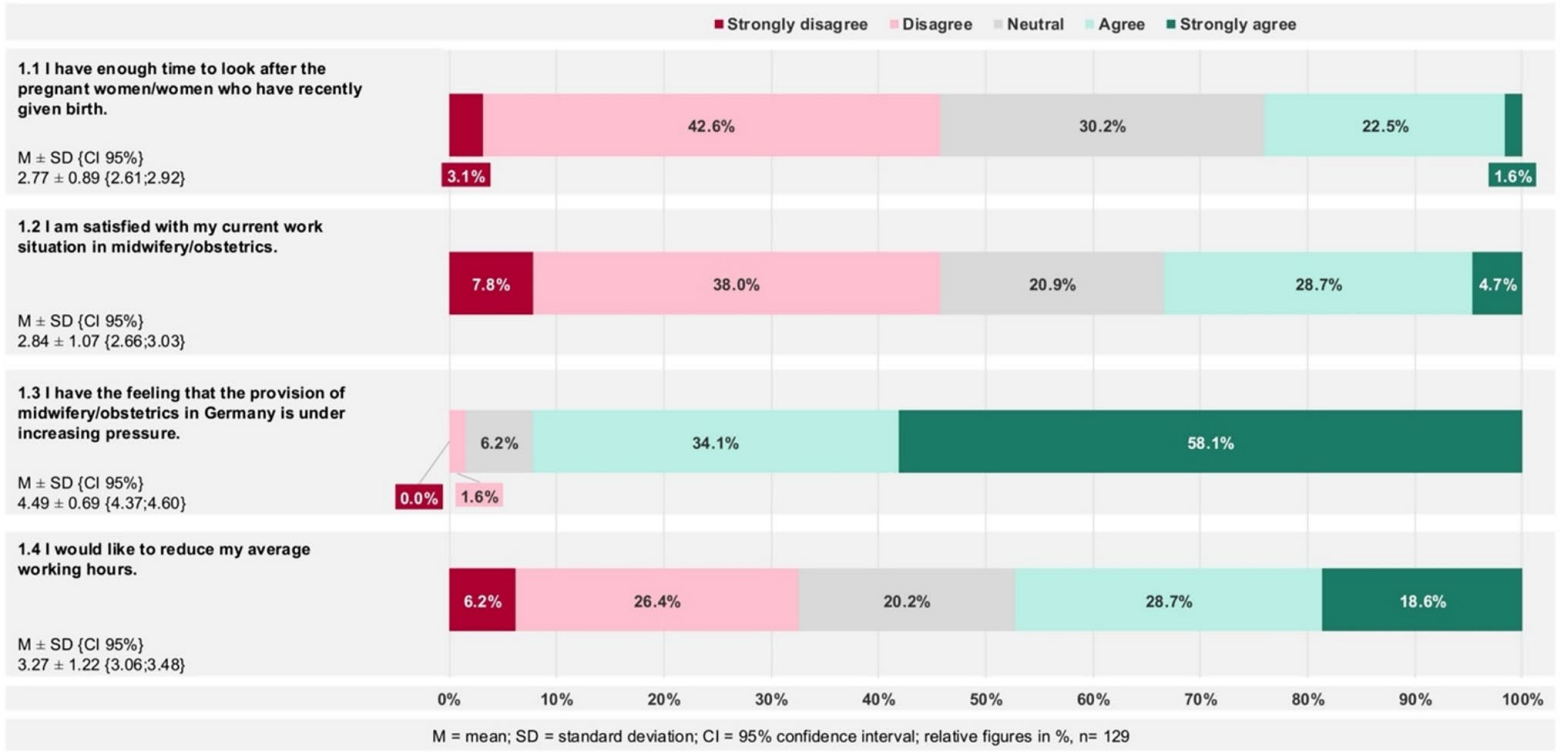
Perception of the current work situation

A total of 47.3% of respondents indicated the desire to reduce working hours (agree and strongly agree; Item 1.4; M = 3.27, SD = ± 1.22, CI = {3.06; 3.48}). Regarding overall satisfaction with their current work situation in midwifery and obstetrics, 33.4% expressed agreement (Item 1.2; 2.84 ± 1.07 {2.66; 3.03}). 24.1% reported having sufficient time to care for pregnant individuals (Item 1.1; 2.77 ± 0.89 {2.61; 2.92}). The highest level of agreement was observed for the statement concerning increasing pressure on midwifery and obstetric care in Germany, with 92.2% (thereof 58.1% strong agreement; Item 1.3; 4.49 ± 0.69 {4.37; 4.60}) (Fig. 2).

### Use of digital tools

**Fig. 3:**
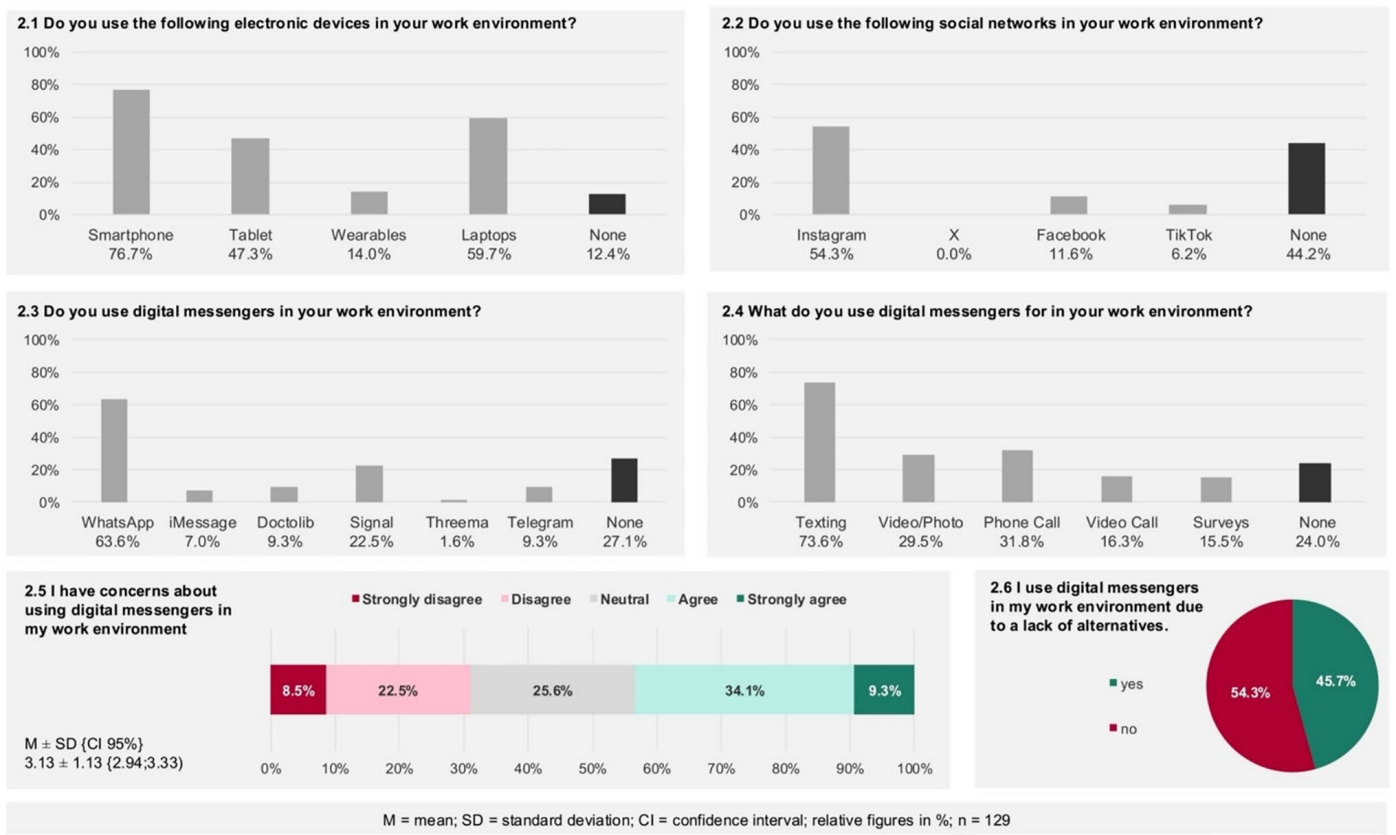
Use of digital tools in work environment

Most respondents reported using digital tools in their professional environment (87.6%). Digital messengers are utilized by 72.9% of the participants and social media platforms by 55.8%. Smartphones are the most commonly used digital device overall (76.7%). Among digital messengers, WhatsApp is the most frequently used, reported by 63.6% of respondents. Messengers are primarily employed for text communication (73.6%), followed by phone calls (31.8%) and the exchange of videos or photos (29.5%). Regarding concerns about using digital messengers in the work environment, 43.4% of respondents expressed agreement and 31.0% disagreement (Item 2.5; 3.13 ± 1.13 {2.94; 3.33}. Furthermore, 45.7% stated to use digital messengers in their work environment due to a lack of alternatives (Fig. 3).

### Perception of digital maternity care

**Fig. 4:**
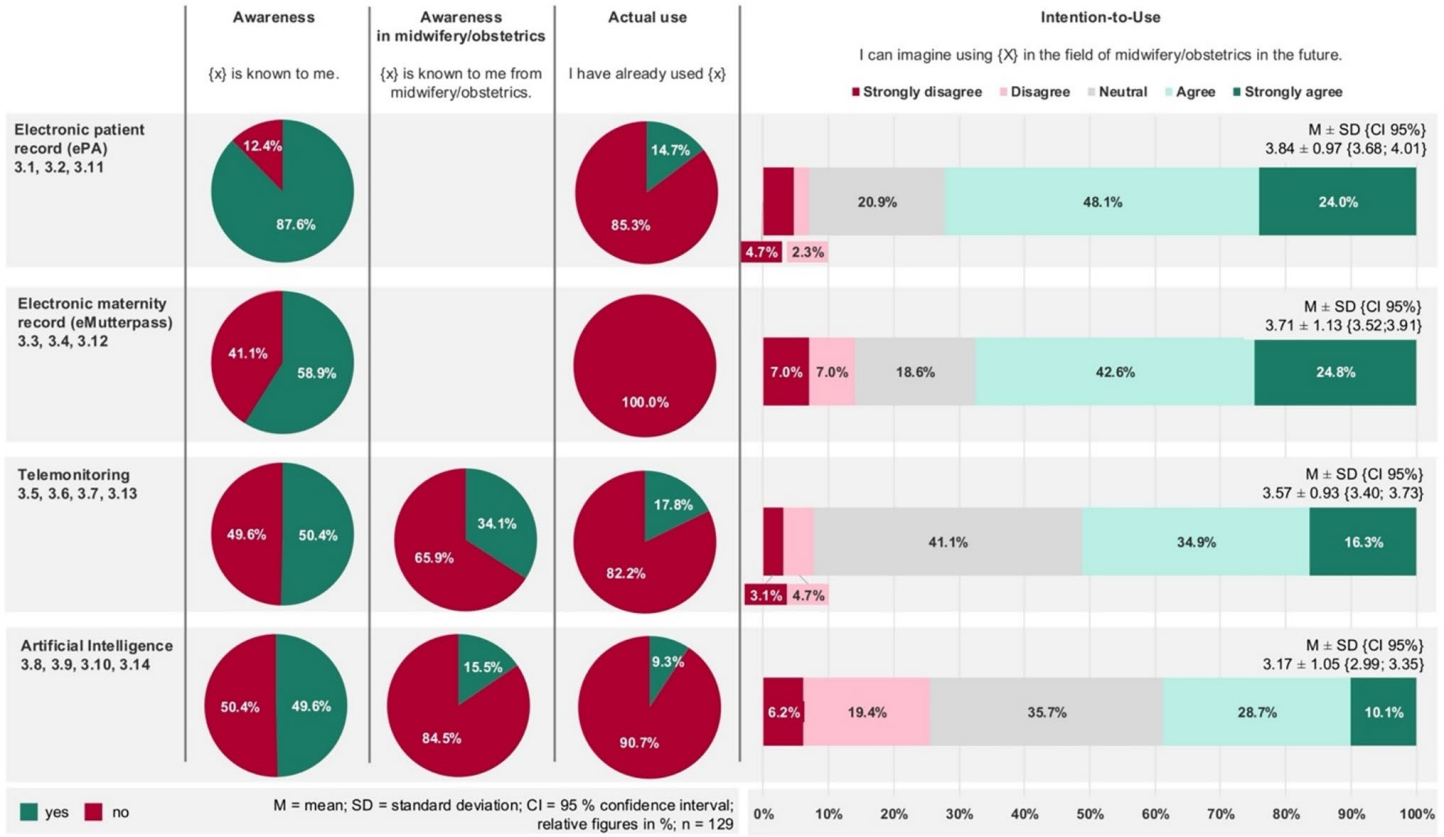
Awareness, intention-to-use and actual use of digital pregnancy care

Awareness of the specific digital health tool decreases from the electronic patient record (ePA; Item 3.1; 87.6%), electronic maternity record (eMutterpass; Item 3.2; 58.9%), telemonitoring (TM) (Item 3.3; 50.4%), to artificial intelligence (AI) (Item 3.4; 49.6%). Awareness on the specific application of TM and AI in midwifery and obstetrics is even lower. Reported actual use is highest for TM (Item 3.7; 17.8%), while no respondents indicated having ever used the eMutterpass. The highest agreement on the intention-to-use can be observed for ePA (72.1%; Item 3.11; 3.84 ± 0.97 {3.68; 4.01}), followed by eMutterpass (67.4%; Item 3.12; 3.71 ± 1.13 {3.52; 3.91}), TM (51.2%; Item 3.13; 3.57 ± 0.93 {3.40; 3.73}), and AI (38.8%; Item 3.14; 3.17 ± 1.05 {2.99; 3.35}). Participants rate their self-perceived digital competence as high (Item 3.14; 7.09 ± 1.48, 95%, {6.83; 7.34}, while also expressing a perceived need for digital training (Item 3.15; 6.03 ± 2.41 {5.61; 6.45}) as measured on a 10-point NRS (Fig. 4).

## Discussion

To date, to our knowledge, no comprehensive study has examined midwives’ and midwifery students’ views on current working conditions, digital tool use, and their awareness and intention-to-use emerging digital solutions in maternity care. This holistic stakeholder analysis approach is, however, a critical determinant of success in digital health initiatives [13]. This study closes this gap, highlights the increasing strain on maternity care and underlines the importance of including midwives as central service providers in the Participatory Design process of a digital maternity care program, as discussed in detail in the following.

### Work situation

The results underline an increasing strain on the care situation as experienced by the midwifery professionals. There is almost complete agreement in the sample, that care is under growing pressure. Overall satisfaction with the work situation was moderate, only about a quarter of the surveyed professionals reported sufficient time to care for pregnant individuals and the majority expressed the desire to reduce working hours. These findings mirror current trends within the German healthcare system for the field of maternity care. Current strain on the system has resulted in a multi-billion-euro deficit among German statutory health insurance providers, prompting both an increase in contribution rates and the need for federal subsidies in the current fiscal year [30]. According to the Federal Ministry of Health, one of the core causes of this situation is the persistent lack of modernization in healthcare structures and care delivery models [31]. If this trend continues unaddressed, it may contribute to the attrition of essential human resources, as professionals may seek alternative employment opportunities outside the maternity care or even the healthcare sector. On international level, digital medicine and eHealth in maternity care have evidenced to provide benefits including cost savings and cost-effectiveness while improving care [5, 32–35]. As such, the increased digitization of maternity care may offer a viable solution to alleviate this strained situation.

### Use of digital tools

The study showcases that a variety of digital tools are already in regular use by the surveyed midwives and midwifery students in their work environment. Even social media platforms have been adopted by half of the respondents in their professional work. While some studies have explored digital tool usage, this study offers one of the first comprehensive overviews within the German context. Recent reviews offer a broad overview of the technologies employed in midwifery settings, but they did not identify studies with a comparable research focus [32, 35–38]. Regarding the messengers being used, the majority relies on commercial providers like WhatsApp™ (WhatsApp Inc. / Meta Platforms, Menlo Park, California, USA), without specific authorization and certification in the medical field. However, since the exchange of photos and videos is one of the core functions in addition to the simple text exchange, a critical question of data security arises. Not only the exchange of sensitive information about pregnancy and the puerperium via text, but above all the exchange of visual data, poses a clear problem. Especially during pregnancy, this often involves very intimate content. This aligns with the finding that the majority of respondents express concerns about relying on the current, oftentimes unofficial, solutions, primarily due to the lack of alternatives. While previous studies have shown that pregnant individuals and physicians generally hold positive attitudes toward digital health, the surveyed midwives and midwifery students tend to express more ambivalent views [36]. Van den Heuvel et al.’s review noted high satisfaction rates among patients using eHealth technologies, ranging up to 95% [35]. Hertle et al.’s study found that over 80% of mothers positively rated digital midwifery services [33]. Nevertheless, as summarized in Vickery et al.’s review on mHealth and eHealth in pregnancy, the surveyed midwives and midwifery students often adopt a more critical view. While they acknowledge the potential of digital tools, they raise important questions [20]. Maternity care is based on interpersonal exchange with a holistic and salutogenetic approach as well as sensation such as smell, sight and touch. Safe digital solutions could provide a first identification and assessment via text, voice, picture and video exchange, while further assessment should be made personally. This study extends this finding to the German context using data from a convenience sample. Previous studies frequently attribute the critical perspective to the strong ethical standards that underpin the midwifery profession [39].

### Perception of digital maternity care

Previous research on digital health in midwifery or obstetrics mostly focused the assessment on singular technologies, e.g., internet use for information seeking, SMS- or app-based support or telemedicine for glucose monitoring in pregnancy [32, 35, 36, 40]. In contrast, this study simultaneously assessed different technologies, that are at different stages of dissemination. In addition, the emerging role of AI in healthcare calls for its inclusion in scientific evaluation, an area largely unexplored in digital maternity care [41]. The study showcases that the intention-to-use decreased in line with the awareness of the various technologies surveyed. While better-known healthcare technologies such as electronic patient records showed higher intention-to-use, the picture is the opposite for newer technologies such as AI. Overall, the agreement on intended use was higher than disagreement for any investigated technology. Nevertheless, actual use rates remained low.

During the COVID-19 pandemic, digital midwifery services expanded in Germany due to lack of alternatives. The majority of mothers who accessed these services, provided by health insurance companies, expressed satisfaction with the digital care options [33]. Despite these encouraging experiences, these digital services were discontinued following the pandemic. In our sample, self-perceived digital competence was relatively high and intention-to-use certified digital technologies generally exceeded disagreement across all four modalities examined (ePA, eMutterpass, TM, AI), although actual use of certified tools remained low. This pattern may indicate that, for the surveyed midwives and midwifery students, barriers to wider adoption are not solely located at the level of individual skills or attitudes. Instead, organizational and policy-level factors, such as reimbursement structures, availability of certified solutions, institutional infrastructure, and data protection requirements, may play an important role. However, our cross-sectional, descriptive design does not allow us to empirically test this hypothesis, and further research explicitly targeting structural determinants is needed. Nevertheless, the findings underscore the importance of including midwives in the implementation process of digital technologies into maternity care, aligning with Vickery et al.’s and Moulaei et al.’s conclusions for careful and targeted rollout strategies [32, 36].

### Limitations

This study has several important limitations. First, although the sample size exceeds previous studies in this field, e.g., Grassl et al. with 36 or Lanssens et al. with 52 midwives, it remains modest relative to the approximately 27,000 midwives in Germany [18, 36]. The use of a convenience sample recruited at a professional congress and through digital networks likely resulted in selection bias, favoring midwives and midwifery students who are professionally networked and potentially more interested in digital health. The high proportion of midwifery students (48.1%) further limits the generalizability of the findings to the practicing midwifery workforce.

Second, the exclusive use of a web-based questionnaire may have privileged individuals who are comfortable with digital tools and have reliable internet access, which could lead to the overestimation of digital competence and acceptance.

Third, only fully completed questionnaires were included in the analysis, and we were unable to characterize those who discontinued the survey. This may introduce attrition bias, as non-completers might have experienced different levels of workload, digital stress, or digital competence.

Fourth, the cross-sectional design precludes causal inferences and does not allow conclusions about temporal changes in attitudes or practices.

Fifth, the questionnaire itself has not undergone formal psychometric validation. Several constructs, such as digital competence and need for training, were operationalized using single, self-developed items. These measures should therefore be interpreted with caution, as they may not fully capture the complexity of the underlying constructs or meet established standards of measurement reliability.

Finally, our analyses were descriptive only, without inferential testing or multivariable modeling. As a result, we cannot formally assess associations between variables, e.g., between perceived workload and intention to use or adjust for potential confounders.

Taken together, these limitations underline that the present findings would be regarded as exploratory and hypothesis-generating, providing an initial needs assessment for a subsequent PD process rather than a representative overview of all midwives in Germany.

### Avenues for future research and participatory design

To address these limitations, future phases of the ongoing research in this project outlined above (see Fig. 1) will incorporate qualitative interviews to gain deeper insights into midwives’ perceptions and contextual experiences with digital tools. Repeated surveys will allow for probabilistic sampling and causal inference, therefore supporting more robust conclusions. In addition, further studies will include other stakeholder groups, such as physicians, nurses and pregnant individuals to provide a more comprehensive view of digital care integration. A planned early clinical feasibility study will generate real-world data on usability, acceptance and implementation further strengthening the evidence. This mixed-methods approach will enhance the depth and generalizability of the current findings.

## Conclusion

This exploratory study provides initial insights into the work environment, use of digital tools and attitudes toward digital health among a non-probabilistic convenience sample of midwives and midwifery students in Germany. Against the backdrop of a perceived strain on maternity care, participants reported substantial use of digital tools, often uncertified ones, and positive intentions to use certified solutions. Self-perceived digital competence was high, while a considerable need for further training was also expressed. These findings suggest that, in this sample, there is openness toward digitally supported maternity care and a willingness to engage with innovation. At the same time, the low reported use of certified digital tools indicates that structural and organizational factors are likely to be critical for successful implementation. Integrating midwives’ perspectives into the design, evaluation and rollout of digital maternity care models appears essentials. As the first step of a broader participatory design process, this study provides an empirical foundation for subsequent qualitative work, iterative prototype development, and early feasibility testing.

## Supplementary Information

Below is the link to the electronic supplementary material.

Supplementary file1 (DOCX 19 KB)

## Data Availability

Data is provided within the manuscript or supplementary information files. Further datasets generated during and analyzed during the current study are available from the corresponding author on reasonable request.

## Declarations

### Explanation of AI-assisted technologies in the writing process

During the proofreading of this paper, the authors used GPT4o to improve readability; after using this tool, the authors reviewed and revised the content as needed and take full responsibility for the content of the publication.
