# Supplementary material for: Digital maternity care in Germany: a cross-sectional web-based survey on midwives’ perceptions
Source: Arch Gynecol Obstet. 2026 Jan 20;313(1):56. doi: 10.1007/s00404-025-08239-5 (PMC12819535; doi:10.1007/s00404-025-08239-5)
Supplement: Supplementary file 1 — Supplementary file1 (DOCX 19 KB) [file 404_2025_8239_MOESM1_ESM.docx]

**Supplementary 1**

**Original Questionnaire in German**

Liebe Hebammen, Liebe Hebammenstudierende,

Das Institut für Digitale Medizin der Philipps-Universität Marburg führt zusammen mit der Hebammenwissenschaft der Hochschule Fulda eine Online-Befragung durch.

Ziel ist es, die Einstellung der Hebammen und Hebammenstudierenden zur Digitalen Gesundheit in der Hebammenkunde zu befragen. 

Wir möchten Dich ganz herzlich zu dieser Befragung einladen. Für jede Teilnahme sind wir sehr dankbar. 

Die Befragung ist freiwillig, anonym und kann jederzeit abgebrochen werden. Es werden keine personenbezogenen Daten erhoben, eine Rückverfolgung ist nicht möglich.

Dieser Abschnitt erfasst deine Einschätzung zur aktuellen Arbeitssituation in der Hebammenkunde/Geburtshilfe, insbesondere im Hinblick auf deine Arbeitsbedingungen und den Versorgungsdruck.

Bitte bewerte die folgenden Aussagen. (5-Punkt Likert Skala)

**Item 1.1** Ich habe ausreichend Zeit für die Betreuung der Schwangeren/Wöchnerinnen.

**Item 1.2** Ich bin mit meiner aktuellen Arbeitssituation in der Hebammenkunde/Geburtshilfe zufrieden.

**Item 1.3** Ich habe das Gefühl, dass die Versorgung der Hebammenkunde/Geburtshilfe in Deutschland zunehmend unter Druck steht.

**Item 1.4** Ich würde gerne meine durchschnittliche Arbeitszeit reduzieren.

Nun folgen einige Fragen zur Nutzung digitaler Medien in deinem beruflichen Umfeld.

**Item 2.1** Benutzt du die folgenden elektronischen Geräte in deinem beruflichen Umfeld?

(Mehrfachauswahl möglich, Texteingabe unter „Weitere“ möglich)

- Smartphone (iPhone, Google Phone, Android etc.)
- Tablet (iPad, Galaxy Tab etc.)
- Wearables (Smartwatch wie Applewatch, Fitnessuhren/-armbänder etc.)
- Laptops (z.B. Macbook, SONY, Asus etc.)
- Keine der genannten Optionen trifft auf mich zu
- Weitere (Freitext)

**Item 2.2** Benutzt du folgende soziale Netzwerke in deinem beruflichen Umfeld?

(Mehrfachauswahl möglich, Texteingabe unter „Weitere“ möglich)

- Instagram
- X
- Facebook
- TikTok
- Keine der genannten Optionen trifft auf mich zu
- Weitere (Freitext)

**Item 2.3** Benutzt du digitale Messenger im beruflichen Umfeld?

(Mehrfachauswahl möglich, Texteingabe unter „Weitere“ möglich)

- WhatsApp
- iMessage
- Doctolib
- Signal
- Threema
- Telegramm
- Keine der genannten Optionen trifft auf mich zu
- Weitere (Freitext)

**Item 2.4** Wofür benutzt du digitale Messenger in deinem beruflichen Umfeld?

(Mehrfachauswahl möglich, Texteingabe unter „Weitere“ möglich)

- Textaustausch
- Foto/Videoaustausch
- Telefonate
- Videotelefonate
- Umfragen
- Keine der genannten Optionen trifft auf mich zu
- Weitere (Freitext)

**Item 2.5** Bitte bewerte folgende Aussage. (5-Punkt Likert Skala)

Ich habe Bedenken digitale Messenger in meinem beruflichen Umfeld zu nutzen.

**Item 2.6** Ich benutze digitale Messenger in meinem beruflichen Umfeld aufgrund von fehlenden Alternativen. (Binär, ja oder nein)

Im folgenden Abschnitt geht es um deinen Kenntnisstand zu Begriffen und Konzepten der digitalen Gesundheit.

Bitte beantworte folgende Fragen. (Binär, ja oder nein)

**Item 3.1** Die elektronische Patientenakte (ePA) ist mir bekannt.

**Item 3.2** Ich habe die elektronische Patientenakte (ePA) bereits verwendet.

**Item 3.3** Der elektronische Mutterpass (eMutterpass) ist mir bekannt.

**Item 3.4** Ich habe den elektronischen Mutterpass (eMutterpass) bereits verwendet.

**Item 3.5** Das Konzept des Telemonitorings ist mir aus anderen Gesundheitsbereichen (beispielsweise dem Herzinsuffizienzmonitoring in der Kardiologie) bekannt.

**Item 3.6** Das Konzept des Telemonitorings ist mir aus der Hebammenkunde/Geburtshilfe bekannt.

**Item 3.7** Ich habe Telemonitoring in der Hebammenkunde/Geburtshilfe bereist verwendet.

**Item 3.8** Die Anwendung von Künstlicher Intelligenz ist mir aus anderen Gesundheitsbereichen (beispielsweise dem Hautkrebsscreening in der Dermatologie) bekannt.

**Item 3.9** Die Anwendung von Künstlicher Intelligenz ist mir in der Hebammenkunde/ Geburtshilfe bekannt.

**Item 3.10** Ich habe Künstliche Intelligenz in der Hebammenkunde/Geburtshilfe bereits verwendet.

Bitte bewerte folgende Aussagen. (5-Punkt Likert Skala)

**Item 3.11** Ich kann mir vorstellen die <u>elektronische Patientenakte (ePA) im Bereich der Hebammenkunde/Geburtshilfe in Zukunft zu verwenden.

**Item 3.12** Ich kann mir vorstellen den <u>elektronischen Mutterpass (eMutterpass) im Bereich der Hebammenkunde/Geburtshilfe in Zukunft zu verwenden.

**Item 3.13** Ich kann mir vorstellen Telemonitoring im Bereich der Hebammenkunde/Geburtshilfe in Zukunft zu verwenden.

**Item 3.14** Ich kann ich mir vorstellen Künstliche Intelligenz in Zukunft im Bereich der Hebammenkunde/Geburtshilfe zu verwenden.

**Item 3.15** Wie bewertest du deine eigene digitale Kompetenz ein auf einer Skala von 1 bis 10?

**Item 3.16** Wie bewertest du den Schulungsbedarf deiner digitalen Kompetenz auf einer Skala von 1 bis 10?

Zum Schluss möchten wir einige demographische Angaben erfassen.

**Item 4.1** Wie alt bist du? (Bitte tippe dein Alter in Jahren ein)

**Item 4.2** Welchem Geschlecht ordnest du dich zu? (Non-binär, männlich oder weiblich)

**Item 4.3** Bitte wähle deine passende Berufsbezeichnung.

- Leitende Hebamme
- Hebamme
- Hebammenstudierende(r)
- Hebamme mit Praxisanleiterschein
- Advanced Practice Midwife (APM)

**Item 4.4** Wie viele Jahre Berufserfahrung hast du seit Abschluss deiner Ausbildung/deines Studiums?

- <2 Jahre
- 2-5 Jahre
- 5-10 Jahre
- 10-20 Jahre
- 20-30 Jahre
- >30 Jahre
- Aktuell noch im Studium

Dies ist die letzte Seite der Umfrage.

Wenn du diese beenden willst dann drücke nun auf "WEITER".

Wenn du deine Antworten weiter bearbeiten willst, dann kannst du den Button "ZURÜCK" nutzen.

Wir möchten uns herzlichst für deine Teilnahme bedanken!

Du kannst die Umfrage nun schließen.
